# Face and content validation of laser enucleation of prostate simulation model: an EAU European School of Urology (ESU) Lower Urinary Tract Endoscopy Working Group study

**DOI:** 10.1007/s00345-026-06428-8

**Published:** 2026-05-06

**Authors:** Tarik Emre Sener, Tiago Ribeiro de Oliveira, Engin Denizhan Demirkiran, Davide Perri, Sergio Pereira, Juan Pablo Caballero, Luis Osorio, Ioannis Goumas Kartalas, Marijn Goossens, Bernhard Schoensee, Laurian Dragos, Domenico Veneziano, Chandra Shekhar Biyani, Bhaskar Somani, Evangelos Liatsikos

**Affiliations:** 1 Department of Urology, School of Medicine, Marmara University, Istanbul, Türkiye; 2 Department of Urology, Armed Forces Hospital, Lisbon, Portugal; 3 Department of Urology, School of Medicine, Zonguldak Bülent Ecevit University, Zonguldak, Türkiye Turkey; 4 Department of Urology, Azienda Socio Sanitaria Territoriale Lariana, Como, Italy; 5 Department of Urology, Lisbon Medical Academic Centre, Lisbon, Portugal; 6 Department of Urology, University General Hospital, Alicante, Spain; 7 Department of Urology, Lusíadas Porto Hospital, Porto, Portugal; 8 Department of Urology, Istituto Clinico Beato Matteo, Vigevano, Italy; 9 Department of Urology, OLV Hospital Aalst, Aalst, Belgium; 10 Department of Urology, Urologie Berlin Adlershof, Berlin, Germany; 11 Department of Urology, Cambridge University Hospitals NHS Foundation Trust, Cambridge, UK; 12 Department of Urology, Smith Institute for Urology, Northwell Health, New York, USA; 13 Department of Urology, St James’s University Hospital, Leeds, UK; 14 Department of Urology, University Hospital Southampton NHS Foundation Trust, Southampton, UK; 15 Department of Urology, University Hospital Patras, Patras, Greece

**Keywords:** prostate, education, laser, enucleation, simulation, validation

## Abstract

**Introduction:**

Laser enucleation of the prostate (LEP) has become a standard treatment for benign prostatic obstruction, yet its adoption is limited by a steep learning curve and a lack of validated training models. This study aimed to evaluate the face and content validity of a bench-top simulator specifically developed for LEP training within the framework of the European School of Urology (ESU) Lower Urinary Tract Endoscopy Working Group.

**Methods:**

Fourteen expert endourologists assessed the simulator during the European Urology Residents Education Programme (EUREP) 2025. Face and content validity were evaluated using 4-point Likert questionnaires, structured using the ESU validation framework. Descriptive statistics and content validity indices (I-CVI, S-CVI/Ave, S-CVI/UA) were calculated to assess agreement across anatomical, procedural, and educational domains.

**Results:**

Face validity scores were high across all items (mean 3.57–3.86; ≥90% agreement). The overall S-CVI/Ave was 0.85 and S-CVI/UA 0.47. Domain-specific analysis showed strong content validity for procedural steps (S-CVI/Ave = 0.89) and educational/global domains (0.95), moderate validity for essential anatomy (0.81), and lower ratings for intraoperative conditions (0.69), primarily due to the absence of bleeding simulation. When restricted to core domains (procedural + educational), S-CVI/Ave improved to 0.91.

**Conclusion:**

The simulator demonstrated strong face and content validity. Expert ratings indicated favorable perceptions of its realism, procedural similarity, and educational utility. These findings suggest that the simulator may be a useful component of a structured transurethral procedural training curriculum, although further construct validation is needed.

## Introduction

Laser enucleation of the prostate (LEP) has been the cornerstone surgical option in the last decade for the management of benign prostatic obstruction for patients with large prostates. The technique, regardless of the energy source used, offers successful symptom relief with low complication rates. Laser enucleation of the prostate has established clinical superiority over transurethral resection techniques, however, widespread adoption of LEP remains limited due to the technical complexities and prolonged learning curve of the technique [1, 2]. Safe skill acquisition requires structured exposure to anatomical recognition, capsular plane dissection, and controlled enucleation maneuvers before progression to live surgery.

Simulation-based training is increasing to overcome these challenges in endourology. Multiple simulators have been validated for transurethral resection of the prostate (TURP) and bladder tumors, contributing significantly to skill development and improving learning curves in daily surgical practice [3–10]. However, studies about bench-top simulators on prostate enucleation remain limited in number, and most available models lack validation. High-fidelity cadaveric and virtual reality simulators have demonstrated value, yet they have limitations due to cost, limited access, and low reproducibility.

To address this gap, a dedicated bench-top simulator for laser prostate enucleation was developed through collaboration between the Max Planck Institute for Medical Research (Heidelberg, Germany) and the European School of Urology (ESU) Lower Urinary Tract Endoscopy Working Group. This model was designed to reproduce key anatomical and procedural elements of enucleation, enabling standardized practice of creating mucosal incisions on the prostate, adenoma-capsule dissection, lobe mobilization, and also morcellation in a reproducible setting.

This study aimed to evaluate the face and content validity of this enucleation simulator based on expert assessments using a standardized validation framework [11, 12]. The primary objective was to determine the model’s realism, procedural fidelity, and educational value as a component of the advanced step of structured transurethral procedural training.

## Methods

### Study design

We conducted a prospective validation study of a bench-top simulator designed for laser enucleation of the prostate. Face and content validity were assessed through structured questionnaires completed independently by expert endourologists. Experts were selected by the European Association of Urology (EAU) based on predefined inclusion criteria, including substantial prior clinical and teaching experience in benign prostatic obstruction surgery and laser enucleation of the prostate. All experts had performed more than 100 BPH procedures and were actively involved in endourological education. A total of 14 experts participated in the assessment and their median annual laser enucleation case volume was 52,5 (10–130) cases per year. The study was conducted during the European Association of Urology Residents Education Programme (EUREP) 2025 in Prague, Czech Republic.

### Ethical considerations

This study did not involve human participants or animal subjects. Ethical approval was therefore not required, as the research consisted solely of expert assessment of a bench-top simulation model for educational validation purposes.

### Training model

**Fig. 1:**
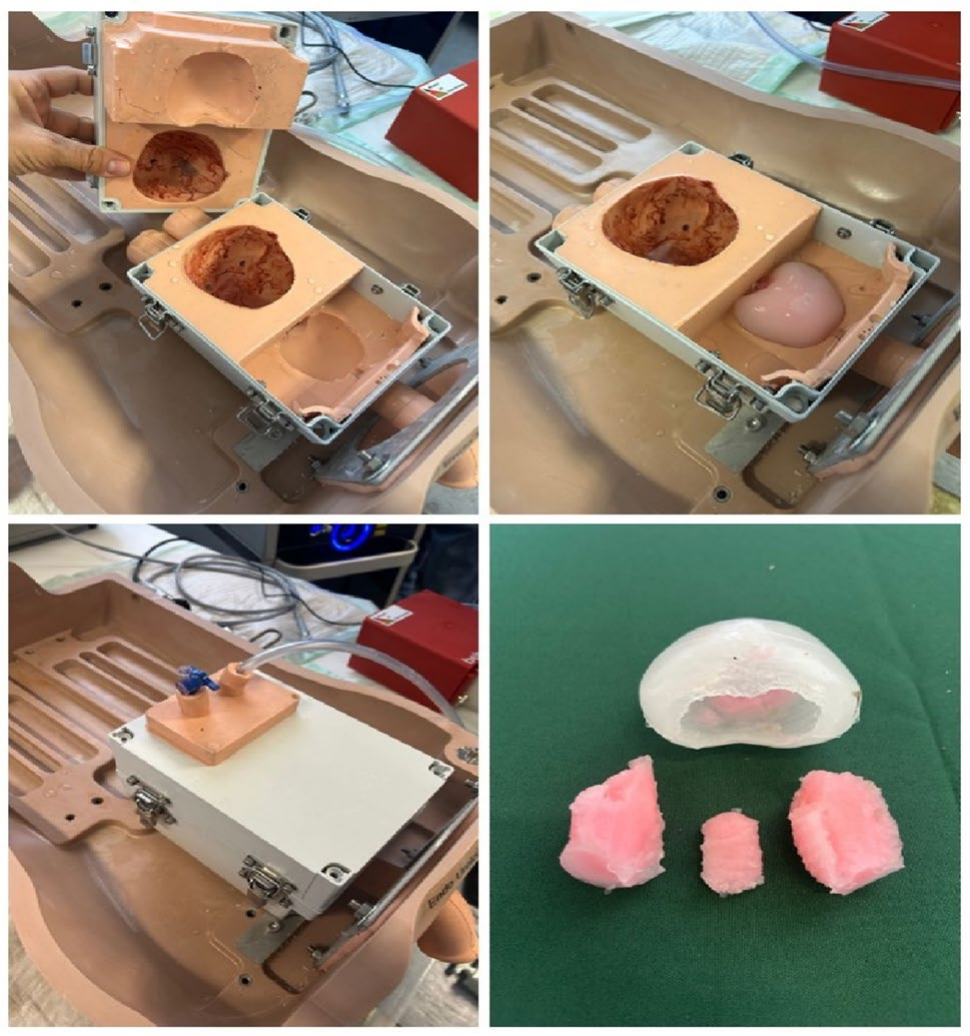
Max Planck Institute enucleation trainer with synthetic prostate module. The box system accommodates a 60 cc prostate model with central lumen and verumontanum

The enucleation training model was developed at the Max Planck Institute for Medical Research as a dedicated simulator for LEP training. The model consists of a compact anatomical box that replicates the bladder and prostatic fossa in a standardized, reusable format. A sealed urethral entry allows insertion of a laser resectoscope. Integrated irrigation tubing permits a fluid-filled environment, while a modular housing unit with a locking mechanism enables both watertight closure and efficient placement and replacement of prostate models (Fig. 1).

### Enucleation module

The replaceable synthetic prostate models are manufactured from soft, non-toxic biomimetic hydrogel materials, which are engineered to match the mechanical properties of normal and BPH tissues to reproduce the tactile feedback and resistance of human prostatic tissue. Ultrasound contrast agents, which are not detectable in endoscopy, are added to distinguish the peripheral zone and the central zone for post-operative quantitative evaluation. Each module incorporates a central urethral lumen with a well-defined verumontanum, two prominent lateral lobes, and a small median lobe, thereby reproducing the key anatomical landmarks necessary for enucleation training. A distinct outer capsule layer to allow trainees to identify and follow the capsular dissection plane, performing both blunt and sharp enucleation maneuvers.

**Fig. 2:**
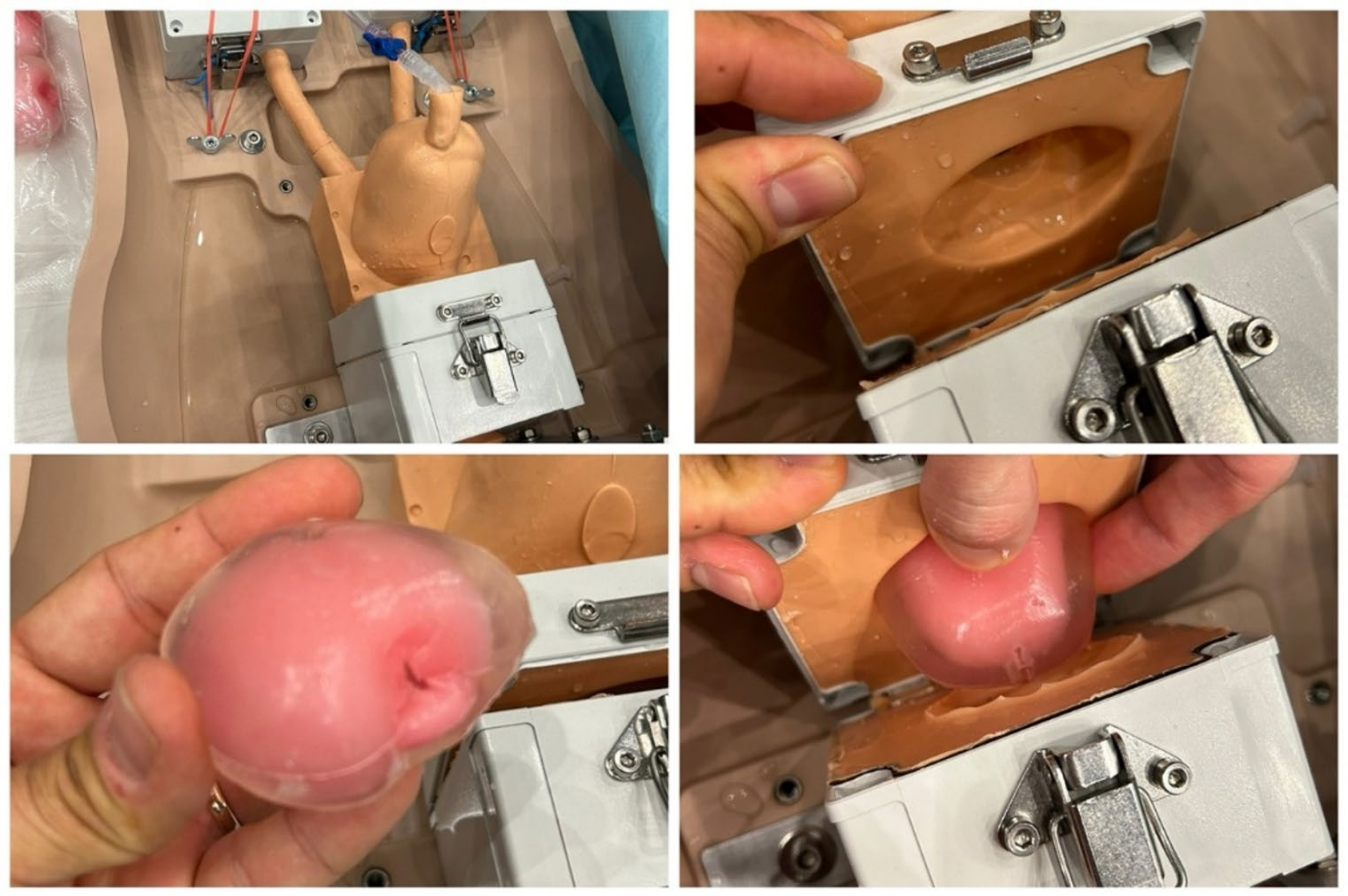
Synthetic prostate modules within the Max Planck trainer. The modular design allows repeated use and quick replacement for standardized laser enucleation of the prostate training

Once mounted into the trainer box, the models can be used to replicate the full sequence of prostate enucleation, including mucosal incision, capsular dissection, lobe mobilization, and morcellation. The modular design permits repeated use and exchange of prostates, ensuring reproducibility and consistency across multiple training sessions (Fig. 2). Ideally, 1 prostate model is used for 1 trainee. For the present study, a 60 cc prostate module was used.

The enucleation training model aims to provide a standardized and reproducible platform for learning the essential steps of prostate enucleation in a controlled setting. Unlike animal or cadaveric models, it offers an ethically sustainable and logistically accessible alternative, allowing repeated practice without the variability of biological tissue. By replicating the main anatomical landmarks and the capsular plane, the model would help enable trainees to safely acquire procedural skills before progression to the operating room.

### Face and content validation questionnaires

The Face and Content Validation Questionnaires for the laser prostate enucleation simulator were designed by the Lower Urinary Tract Endoscopy Working Group of the European School of Urology. These instruments were adapted from the group’s standardized framework for evaluating transurethral training models.

### Face validation

Face validation was assessed using a 9-item questionnaire adapted from previous simulation validation studies and tailored to enucleation training [12–14]. Each item was rated on a 4-point Likert scale (1 = Strongly Disagree, 2 = Disagree, 3 = Agree, 4 = Strongly Agree). Domains included anatomical realism, tissue handling, enucleation plane representation, visibility and access, teaching ergonomics, practice of challenging steps (e.g., capsular plane identification, lobe separation), sequence of surgical steps, training applicability, and overall realism.

Descriptive statistics (mean, median, standard deviation, and range) were calculated for each item. The percentage of agreement was defined as the proportion of ratings ≥ 3. Face validity was considered high if the median score was ≥ 3 and agreement exceeded 75%.

### Content validation

Content validation was evaluated with a 15-item questionnaire addressing key anatomical landmarks, procedural steps, intraoperative conditions, and global educational value of the model. Each item was rated on a 4-point relevance scale (1 = Not Relevant, 2 = Somewhat Relevant, 3 = Quite Relevant, 4 = Highly Relevant).

For each item, the Item-Level Content Validity Index (I-CVI) was calculated as the proportion of experts assigning a rating of 3 or 4, indicating that the item was considered relevant. Scale-level content validity was assessed using two complementary methods: the average approach (S-CVI/Ave), calculated as the mean of the I-CVI values across all items, and the universal agreement approach (S-CVI/UA), calculated as the proportion of items that achieved a rating of 3 or 4 by all experts.

The indices were calculated as follows:

- I-CVI = (number of experts rating an item as 3 or 4)/(total number of experts).
- S-CVI/Ave = (sum of I-CVI values across all items)/(total number of items).
- S-CVI/UA = (number of items achieving universal agreement among experts)/(total number of items).

In line with established recommendations from previous studies, at the item level, an I-CVI value of ≥ 0.78 was considered acceptable. At the scale level, content validity was summarized using both S-CVI/Ave and S-CVI/UA. Because the universal agreement method is more conservative, particularly with larger expert panels, the predefined benchmark for excellent scale-level content validity was based on S-CVI/Ave, with values ≥ 0.90 considered indicative of excellent content validity, while S-CVI/UA was reported as a complementary descriptive measure of complete expert agreement [15, 16].

For interpretability, the 15 items were organized into four domains reflecting distinct aspects of enucleation training:

- Essential Anatomy – ureteric orifices, verumontanum, external sphincter.
- Procedural Steps – incision, capsular dissection, blunt and sharp enucleation, lobe separation, morcellation.
- Intraoperative Conditions – bleeding management, visibility, laser settings.
- Educational/Global – overall coverage of steps, novice/advanced differentiation, overall usefulness.

This structure was chosen to distinguish critical anatomical and procedural elements from secondary intraoperative conditions and educational aspects.

As a secondary sensitivity analysis, a core composite index was also calculated, restricted to procedural and educational/global domains, representing the model’s primary training objectives. Bleeding was excluded, as it is not realistically represented in the model.

### Statistical analysis

Descriptive statistics were used to summarize expert ratings. Analyses were performed using Microsoft Excel (Microsoft Corporation, Redmond, WA, USA).

## Results

### Face validity

**Fig. 3:**
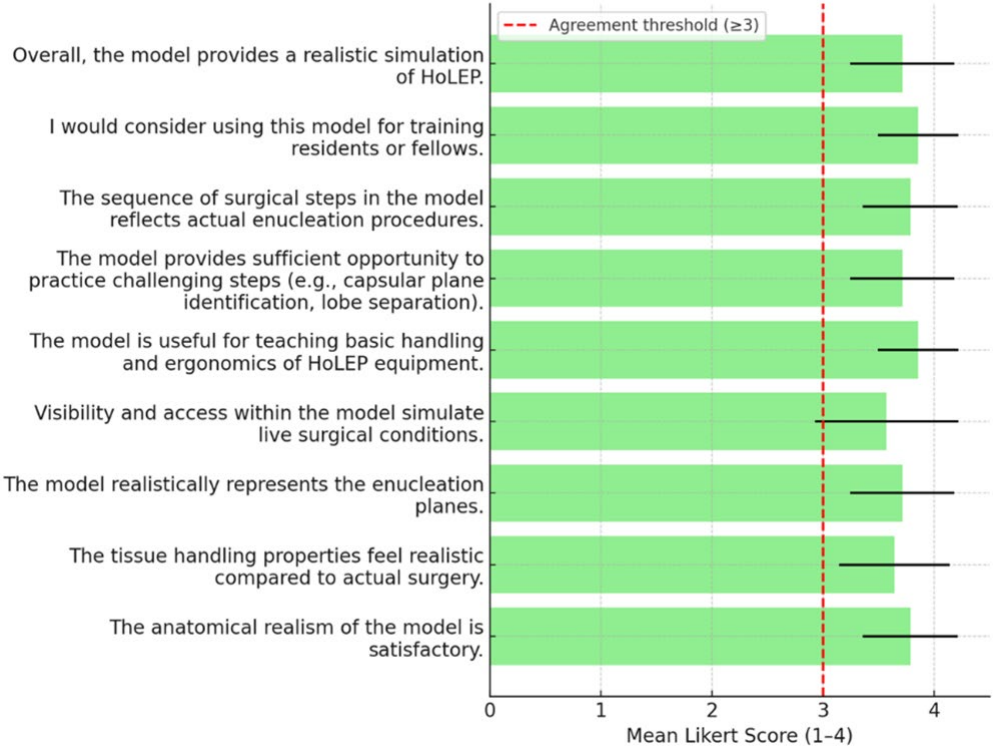
Face validity results of the enucleation training model (*n* = 14). Bars represent mean Likert scores (1–4) with standard deviation error bars. The red dashed line indicates the agreement threshold (≥ 3)

All 14 expert endourologists completed the face validity survey. Responses demonstrated excellent face validity across all domains (Table 1; Fig. 3). Median ratings were consistently 4 (Strongly Agree), with mean scores ranging from 3.57 to 3.86 on a 4-point scale. Agreement rates (ratings ≥ 3) were above 90% for all items, with most domains reaching 100%.

Anatomical realism, tissue handling properties, and representation of enucleation planes were rated highly (means > 3.6, 100% agreement).

Visibility and access conditions received slightly lower ratings (mean 3.57 ± 0.65, agreement 92.9%) but remained above the predefined threshold.

Items regarding ergonomics, challenging steps, surgical sequence, training suitability, and overall realism all achieved 100% agreement.

These results confirm that the model provides a realistic and educational simulation environment for enucleation procedures.

### Content validity

**Fig. 4:**
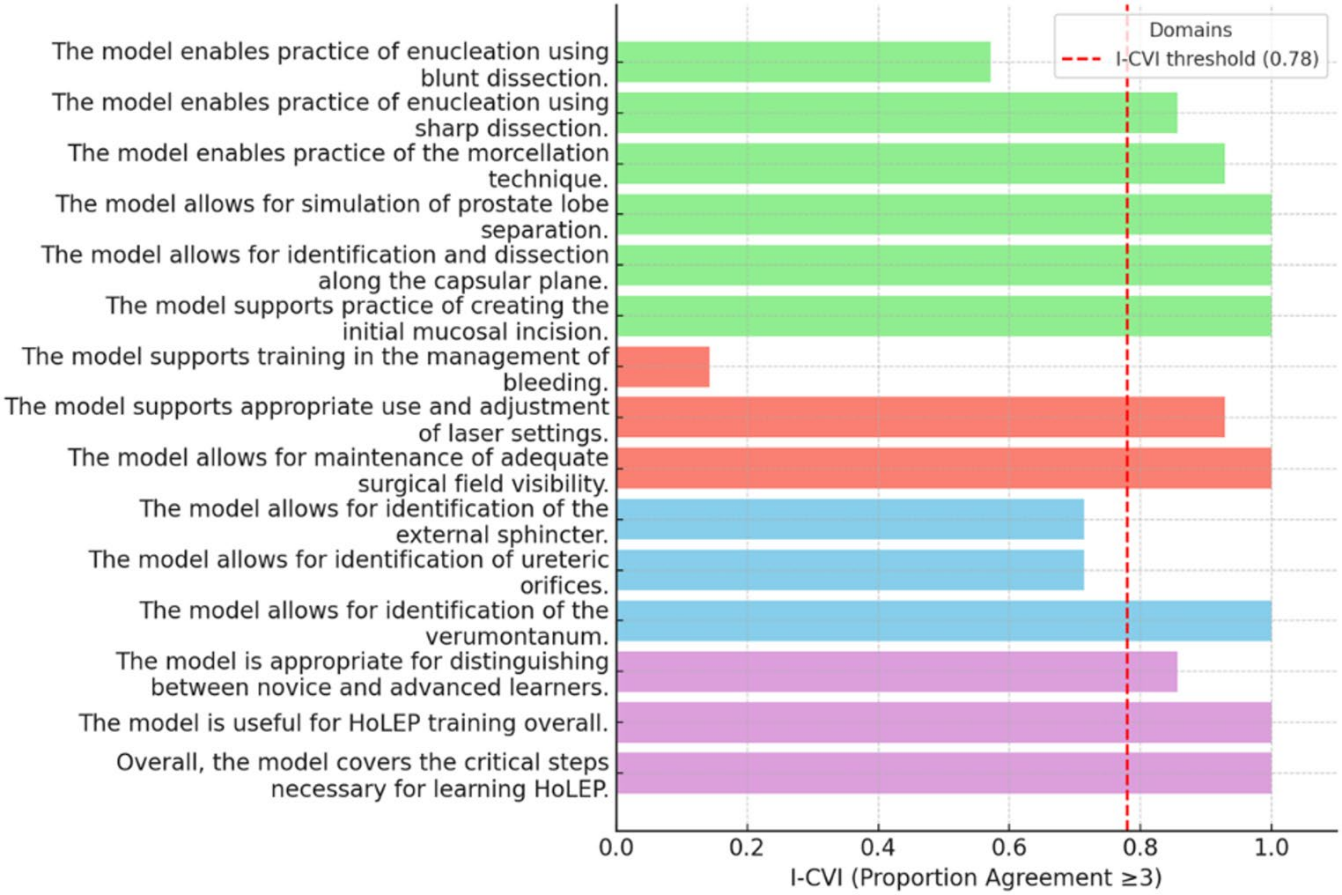
Content validity indices (I-CVI) by item for the enucleation training model (*n* = 14). Horizontal bars represent the proportion of experts rating each item as “Quite Relevant” or “Highly Relevant.” The red dashed line indicates the threshold for excellent content validity (0.78). Items are grouped into four domains: Essential Anatomy, Procedural Steps, Intraoperative Conditions, and Educational/Global

The content validation survey also received 14 expert responses (Table 2; Fig. 4). The overall S-CVI/Ave was 0.85, below the predefined threshold of 0.90 for excellent scale-level content validity, while the S-CVI/UA was 0.47, indicating universal agreement across fewer than half of the items.

Domain-specific analysis provided further insight:

- Procedural steps showed a high level of agreement (S-CVI/Ave = 0.89), reflecting consensus on incision, capsular dissection, enucleation, lobe separation, and morcellation.
- Educational/global items received the highest ratings (S-CVI/Ave = 0.95), exceeding the threshold for excellent scale-level content validity.
- Essential anatomy also showed a high level of agreement (S-CVI/Ave = 0.81), with high ratings for verumontanum identification but lower ratings for ureteric orifice and sphincter identification.
- Intraoperative conditions received the lowest ratings (S-CVI/Ave = 0.69), largely due to poor ratings for bleeding management, which the model does not simulate.

Sensitivity analysis (Core composite): Because the model’s primary purpose is to train the enucleation steps, a restricted analysis was performed including only procedural steps and educational/global items. In this core set, the S-CVI/Ave improved to 0.91, meeting the threshold for excellent scale-level content validity, while S-CVI/UA remained 0.50. These findings suggest that although certain intraoperative aspects (e.g., bleeding) are not realistically represented, the model demonstrates excellent validity for its intended educational purpose.

## Discussion

This study evaluated the educational validity of a bench-top simulator specifically developed for laser prostate enucleation training. Both face and content validation were conducted by expert endourologists from multiple European centers. The results showed favorable expert ratings for anatomical realism and procedural relevance, and supported the simulator’s potential usefulness for structured skills training in enucleation techniques.

While face validity achieved universal agreement among experts, confirming the model’s realism and ergonomic adequacy, content validity varied across specific domains. The strongest consensus was observed for procedural and educational aspects, suggesting that the simulator reproduces several key steps of prostate enucleation and may offer educational value in a simulation setting. In contrast, intraoperative conditions such as bleeding management and sphincter identification received lower ratings, emphasizing the inherent challenges in reproducing these dynamic features in a synthetic model.

Although simulation in transurethral surgery is well established, studies focusing specifically on prostate enucleation remain limited in number [5, 7, 17, 18]. Early validation studies established the foundation for simulation-based enucleation training. Aydın et al. (2014) conducted one of the first validations of a Holmium laser enucleation simulator, confirming its face and content validity and strong user acceptance among both trainees and senior urologists [19]. Shortly after, Kuronen-Stewart et al. (2015) evaluated a virtual reality HoLEP simulator (UroSim™) across multiple centers, demonstrating face, content, and construct validity, with experts outperforming novices [20]. Together, these studies demonstrated that both physical and virtual simulation modalities could reproduce key procedural steps and provide structured learning opportunities. Our model builds on these early efforts by applying a standardized, domain-based validation framework for quantitative assessment of anatomical, procedural, and educational realism.

Subsequent research refined simulator realism and curricular integration. Kallidonis et al. (2021) performed a systematic review of enucleation training models, identifying several with promising validity but limited educational standardization [21]. In the same year, Tunc et al. (2021) validated a fresh-frozen cadaver model for holmium laser enucleation, reporting excellent anatomical realism but limited reproducibility and cost-effectiveness [22]. In contrast, this bench-top simulator may represent a more accessible and reproducible alternative, with positive face and content validity findings, while potentially addressing some of the practical limitations of cadaveric or virtual models.

Recent studies have emphasized structured design and multicentric validation. Shepard et al. (2024) developed a hydrogel-based HoLEP simulator through a Delphi consensus process and demonstrated strong construct validity across multiple centers [23], while Siron et al. (2025) highlighted in their scoping review that most available enucleation simulators still lack standardized validation frameworks [24]. Consistent with these findings, our study provides face and content validation data supporting the potential educational role of a standardized bench-top enucleation model, aligning with ongoing efforts to integrate validated simulators into formal endourology curricula.

**Table 1:** Face validity results of the enucleation training model (*n* = 14)

Item	Mean ± SD	Median	% agreement (≥ 3)
Anatomical realism of the model is satisfactory	3.79 ± 0.43	4	100%
Tissue handling properties feel realistic compared to surgery	3.64 ± 0.50	4	100%
Model realistically represents the enucleation planes	3.71 ± 0.47	4	100%
Visibility and access simulate live surgical conditions	3.57 ± 0.65	4	92.9%
Useful for teaching equipment handling and ergonomics	3.86 ± 0.36	4	100%
Provides opportunity to practice challenging steps	3.71 ± 0.47	4	100%
Sequence of surgical steps reflects actual enucleation	3.79 ± 0.43	4	100%
Suitable for training residents or fellows	3.86 ± 0.36	4	100%
Overall, the model provides a realistic simulation of laser enucleation of the prostate	3.71 ± 0.47	4	100%

**Table 2:** Content validity results of the enucleation training model (*n* = 14)

Item	Mean ± SD	Median	I-CVI	Domain
Identification of ureteric orifices	2.71 ± 1.07	3	71.4%	Essential anatomy
Identification of verumontanum	3.86 ± 0.36	4	100%	Essential anatomy
Identification of external sphincter	3.21 ± 0.89	3	71.4%	Essential anatomy
Initial mucosal incision	3.79 ± 0.43	4	100%	Procedural steps
Capsular plane dissection	3.71 ± 0.47	4	100%	Procedural steps
Enucleation using blunt dissection	2.86 ± 1.17	3	57.1%	Procedural steps
Enucleation using sharp dissection	3.50 ± 0.76	4	85.7%	Procedural Steps
Prostate lobe separation	3.86 ± 0.36	4	100%	Procedural steps
Management of bleeding	1.50 ± 0.94	1	14.3%	Intraoperative conditions
Maintenance of surgical field visibility	3.57 ± 0.51	4	100%	Intraoperative conditions
Laser settings adjustment	3.43 ± 0.65	3	92.9%	Intraoperative conditions
Morcellation technique	3.21 ± 0.80	3	92.9%	Procedural steps
Coverage of critical laser enucleation of the prostate steps	3.79 ± 0.43	4	100%	Educational/global
Distinguishing novice vs. advanced learners	3.43 ± 0.94	4	85.7%	Educational/global
Overall usefulness for laser enucleation of the prostate training	3.86 ± 0.36	4	100%	Educational/global

The findings of this study suggest that prostate enucleation simulators may have a role in structured endourology training curricula. However, before integration into formal urological training curricula, further studies are required to assess construct and predictive validity in broader trainee cohorts. Simulation-based training offers a reproducible and safe environment for skill acquisition, especially for technically demanding procedures such as laser enucleation, which has a recognized learning curve. The validated model presented here can serve as an entry-level platform for residents and early trainees to develop orientation, anatomical recognition, and procedural sequencing skills before advancing to cadaveric or live surgical practice. By providing a controlled, accessible training tool, this simulator may help support early skills development in laser enucleation of the prostate. However, its effect on the learning curve, operative performance, patient safety and training standardization requires further study. In addition, its standardized design and reproducible metrics make it suitable for integration into modular curricula, such as the European School of Urology’s transurethral training framework [25, 26].

While the findings support the simulator’s integration into training curricula, several limitations must be acknowledged. The study assessed validity through expert evaluations only, without including novice or intermediate trainees to measure construct or predictive validity. Consequently, the model’s ability to differentiate performance levels or predict operative competency remains to be established. In addition, the Cognitive Task Analysis (CTA), Delphi consensus process, and metrics development for the simulator-based training pathway have already been completed and will be reported separately. These elements form the methodological basis for a dedicated construct validity study, which will require a separate setup, study population, and analysis framework. Another limitation of this study is the use of a 4-point Likert scale for face and content validity assessment. Although this format is commonly used in CVI-based validation studies as it may facilitate relevance judgments, it does not provide a neutral response option. As a result, the forced-choice structure may have influenced response patterns and may have contributed to more favorable face validity ratings for some items. Furthermore, although the simulator accurately reproduces anatomical and procedural components, dynamic intraoperative factors such as bleeding, irrigation flow, and tissue resistance cannot be fully replicated in a synthetic model and hence, it cannot be used to train decision-making and technical adaptation in response to dynamic intraoperative challenges like significant hemorrhage. Future research should focus on construct and predictive validation phases, including objective performance metrics and longitudinal learning assessment to define its role within a progressive training pathway.

This validation study was done within the framework of the European School of Urology (ESU), reflecting the commitment of EAU to structured, competency-based surgical training [23]. This may support future efforts towards more standardised training in enucleation techniques across different centers [24]. Future multicentric validation through the EAU and national societies may help define how this model could fit within a progressive learning pathway from bench-top simulation to clinical practice, in order to support potentially safer, more efficient and standardised training in laser enucleation of the prostate.

## Conclusion

This study demonstrated favorable face and content validity findings for the prostate enucleation bench-top simulator, with supportive expert ratings for anatomical realism, procedural relevance, and educational utility in an expert-based assessment setting. The model represents a standardized and reproducible platform for practicing key steps of laser enucleation. However, further studies assessing the construct validity in broader trainee cohorts are needed before integration into structured transurethral treatment curricula can be recommended.

## Data Availability

Data can be provided upon request.
